# Whole Breast Invasive Lobular Carcinoma Not Detected Radiographically

**DOI:** 10.7759/cureus.10438

**Published:** 2020-09-14

**Authors:** Panagiotis Vlastarakos, Spyridon Marinopoulos, Catherine Dimopoulou, Constantine Dimitrakakis

**Affiliations:** 1 Obstetrics and Gynecology, National and Kapodistrian University of Athens, Alexandra Hospital, Athens, GRC; 2 Pathology, National and Kapodistrian University of Athens, Alexandra Hospital, Athens, GRC

**Keywords:** breast cancer, invasive lobular carcinoma

## Abstract

Breast cancer is the most commonly occurring cancer in women, with invasive lobular carcinoma being the second most common histologic form. A 78-year-old female patient presented complaining of an enlarged palpable lymph node in the left axilla. Breast ultrasound, digital mammography, and contrast-enhanced spectral mammography (CESM) revealed no abnormal findings. Core needle biopsy of the lymph node revealed infiltrative, diffuse neoplastic growth suggestive of adenocarcinoma, indicating that the primary site should be sought in the breast. The patient underwent mastectomy and the histopathology was suggestive of invasive lobular carcinoma throughout the whole extent of the breast parenchyma. Breast cancer should be definitely included in the differential diagnosis of enlarged axillary lymph nodes, even if there is no other clinical or radiographic presentation of breast disease.

## Introduction

Breast cancer is the most commonly occurring cancer in women and the second most common cancer overall. There were over 2 million new cases in 2018, and it is estimated that approximately 627,000 women died from breast cancer. Invasive lobular carcinoma is the second most common histologic form of breast cancer, representing 5% to 15% of all invasive breast cancers [1]. In roughly 5% of women with breast cancer, metastases are clinically evident at the time of diagnosis [2]. The challenge with invasive lobular carcinomas is that they can be difficult to detect clinically and radiographically. Consequently, diagnosis in advanced stages is not rare.

According to the Surgical CAse REport criteria (SCARE criteria), we report the case of a female patient who presented with an enlarged axillary lymph node as the sole clinical manifestation, normal mammographic and ultrasonic findings, who was ultimately diagnosed with invasive lobular breast cancer.

## Case presentation

A 78-year-old Caucasian female patient presented to the outpatient department of our Breast Unit complaining of an enlarged palpable lymph node in the left axilla, first noticed three months ago. She was a mother of two children, delivered with vaginal birth and breastfed until the age of 12 months. She was a non-smoker; her body mass index (BMI) was 30.1 kg/m2 and her menstrual cycle arrested at the age of 50 years. Her personal history reported high blood pressure under treatment with 5 mg nebivolol hydrochloride and 5 mg amlodipine besylate daily and atrial fibrillation under treatment with sotalol 160 mg, propafenone hydrochloride 150 mg, and 3 mg of acenocoumarol daily. Her family history was positive for cancer since her father was diagnosed with stomach cancer.

Upon arrival, physical examination revealed a sole enlarged, mobile, not tender lymph node in the left axilla, with no palpable masses on both breasts and no palpable lymph nodes in contralateral axilla and supraclavicular regions. The overlying skin was of normal appearance. Laboratory findings were within normal range and infection markers were not elevated.

Breast ultrasound performed with Matrix 9-14 MHz and 4D 6-16 MHz automatic scanning probes revealed a 2.2 cm lymph node of round shape with thickened cortex in the left axilla. On Doppler mode, increased blood circulation was also noticed. Comparing to the ultrasound performed two years ago, the lymph node was increased in size by 1 cm and inner blood circulation was a new finding. No abnormal findings were revealed from the scanning of both breasts.

**Figure 1:**
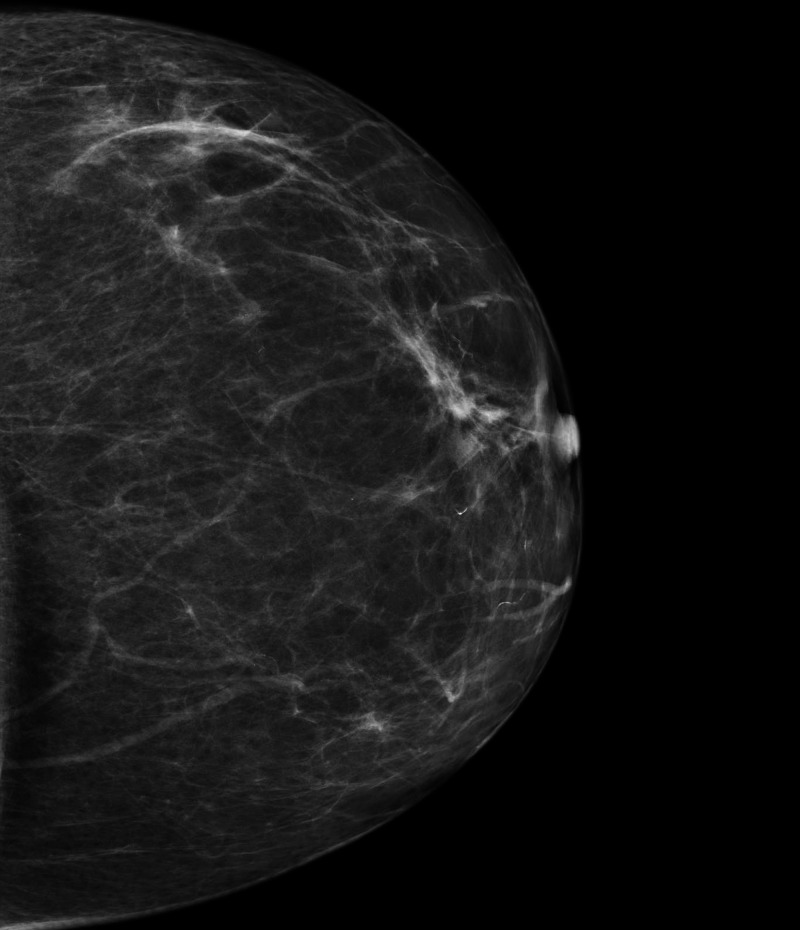
Mammography left craniocaudal view (LCC) demonstrating no abnormal findings

**Figure 2:**
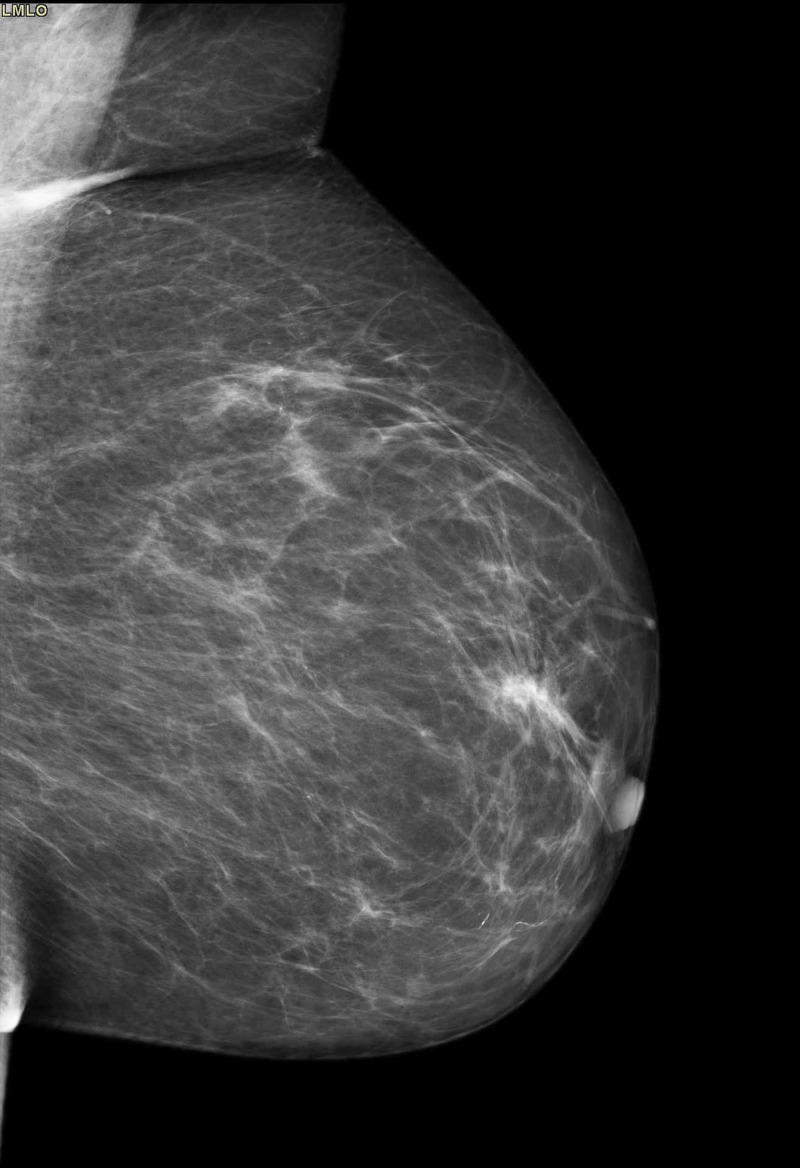
Mammography left mediolateral oblique (LMLO) demonstrating no abnormal findings

On digital mammography, there were no signs indicating malignancy. Left breast presented with normal for the age appearance. Microcalcification at the 10th surgical hour behind the nipple of the contralateral breast were noticed, a finding that was also present and unchanged, compared to the digital mammography performed two years ago, as can be seen in Figure 1 and Figure 2. Contrast-enhanced spectral mammography (CESM) was then performed, providing no additional findings to indicate findings of malignancy.

**Figure 3:**
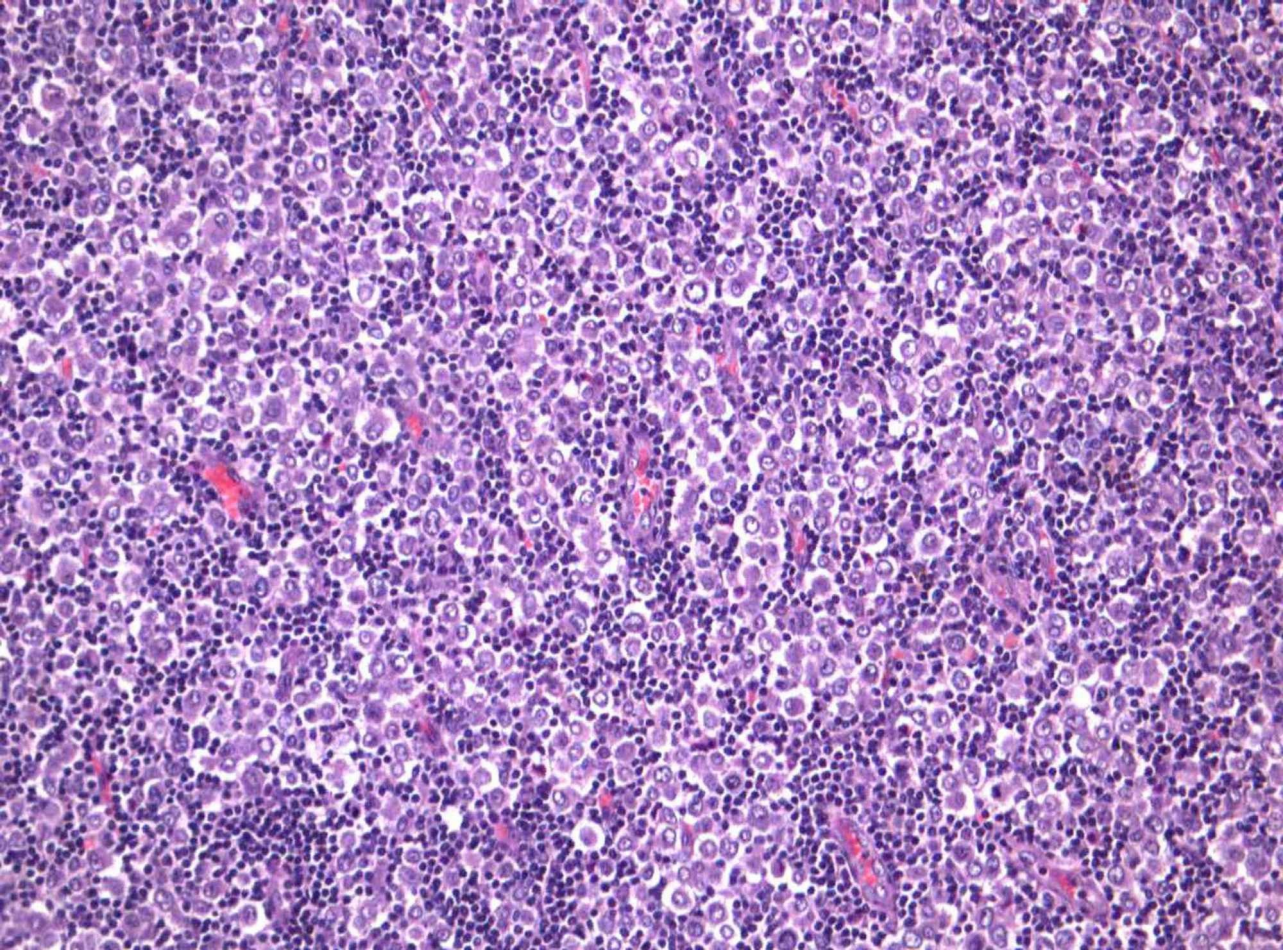
Breast carcinoma of lobular type metastatic to lymph node (HE X200)

**Figure 4:**
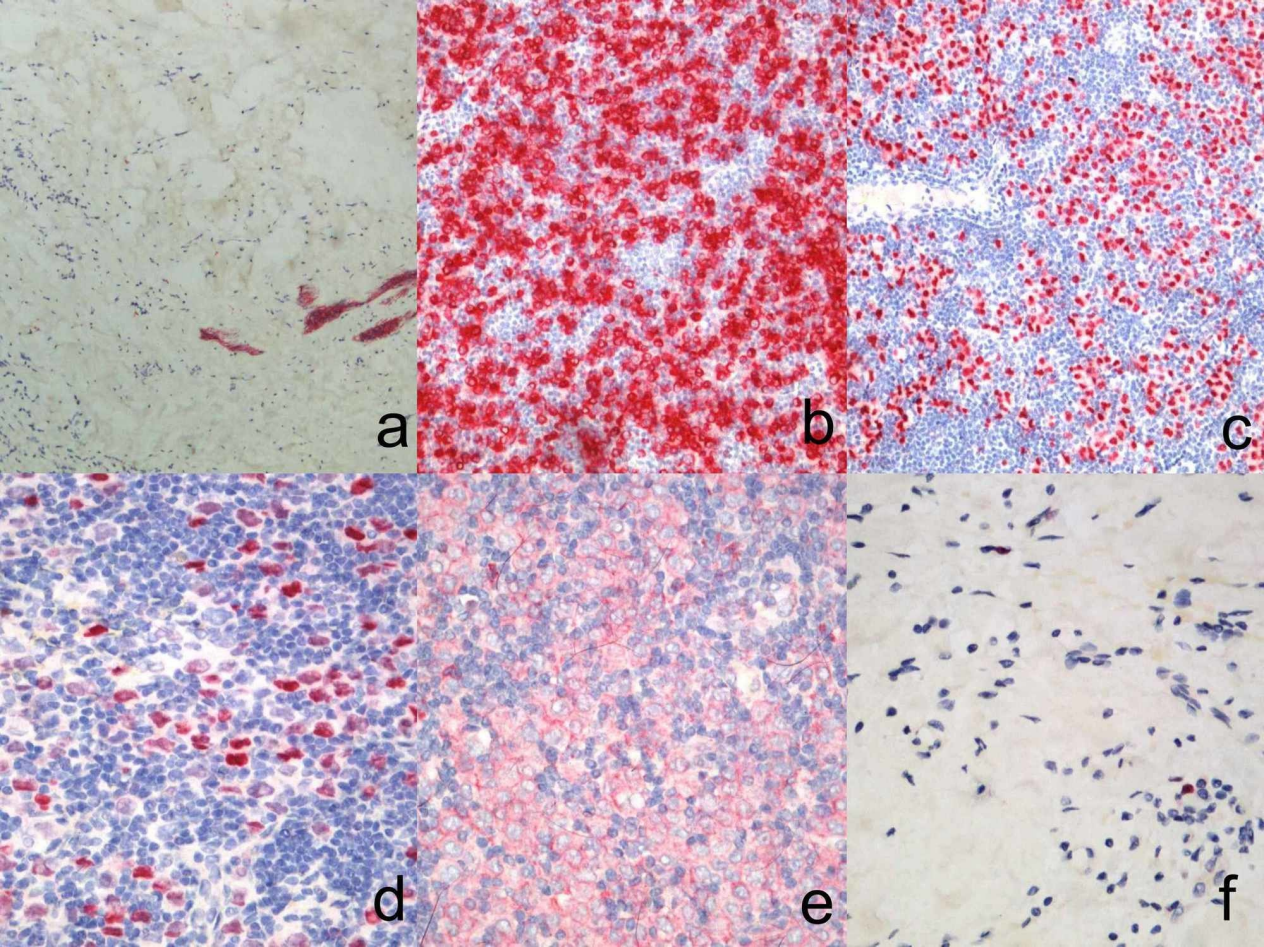
Immunohistochemistry results (HE x200) a) Lack of e-cadherin expression, b) cytokeratin 7 (CK7) positive expression, c) estrogen receptor (ER) positive expression, d) progesterone receptor (PR) positive expression, e) human epidermal growth factor receptor 2 (HER2) negative expression, f) Ki-67 labeling index 40%

The patient underwent core needle biopsy of the lymph node, as the next step of the diagnostic procedure. Histopathological study revealed infiltrative and diffuse neoplastic growth suggestive of adenocarcinoma, indicating that the primary site should be sought in the breast as is demonstrated in Figure 3. Immunohistochemically, the neoplastic cells lacked E-cadherin expression and were positive for cytokeratin 7 (CK7) expression. Estrogen receptor (ER) was expressed in 80% of cells, progesterone receptor (PR) in 60% of cells, human epidermal growth factor receptor 2 (HER2) score was 1+ and Ki-67 labeling index was 40%. Immunohistochemistry results are demonstrated in Figure 4.

**Figure 5:**
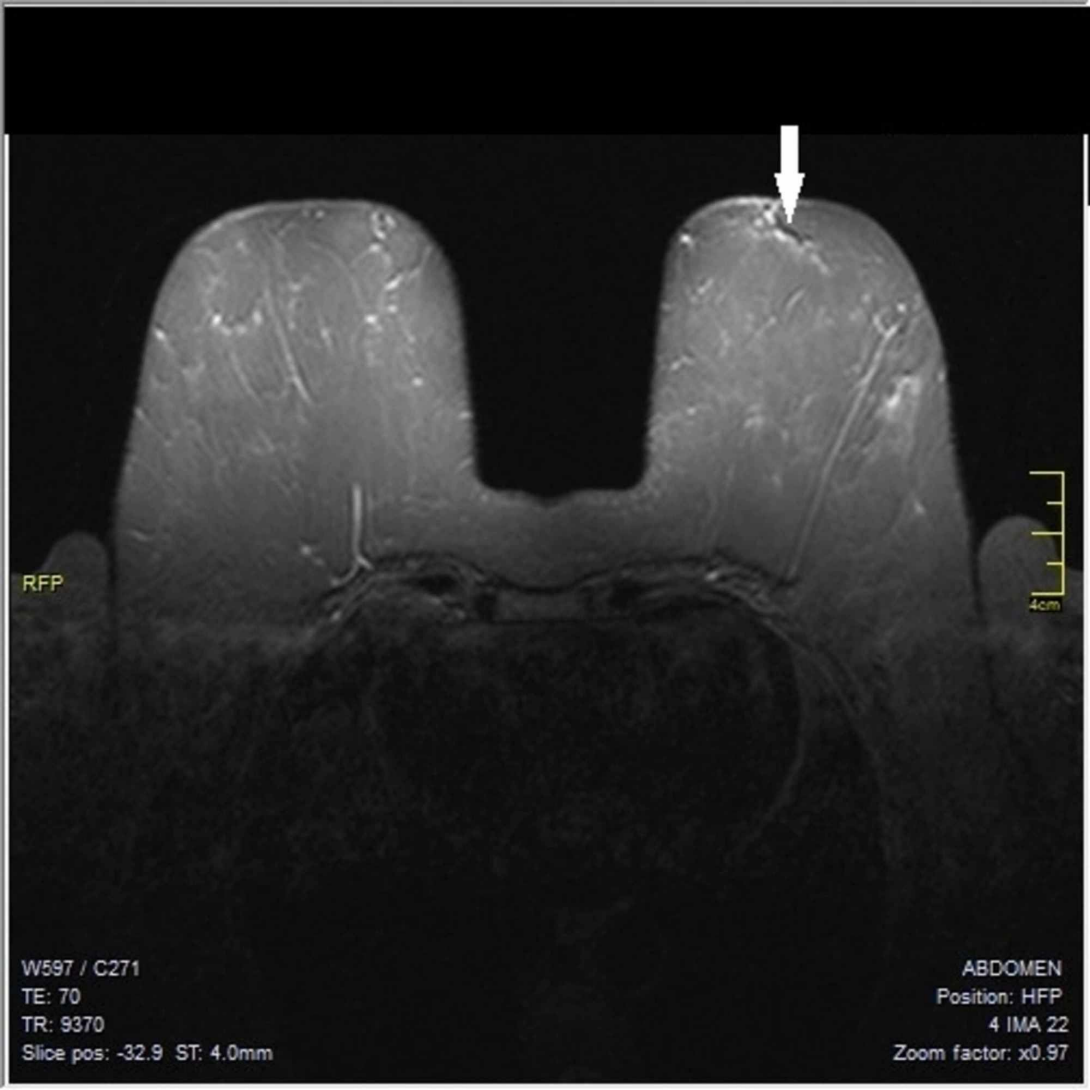
Magnetic resonance mammography (MRM) demonstrating a linear lesion, mammography and ultrasound (US) invisible

**Figure 6:**
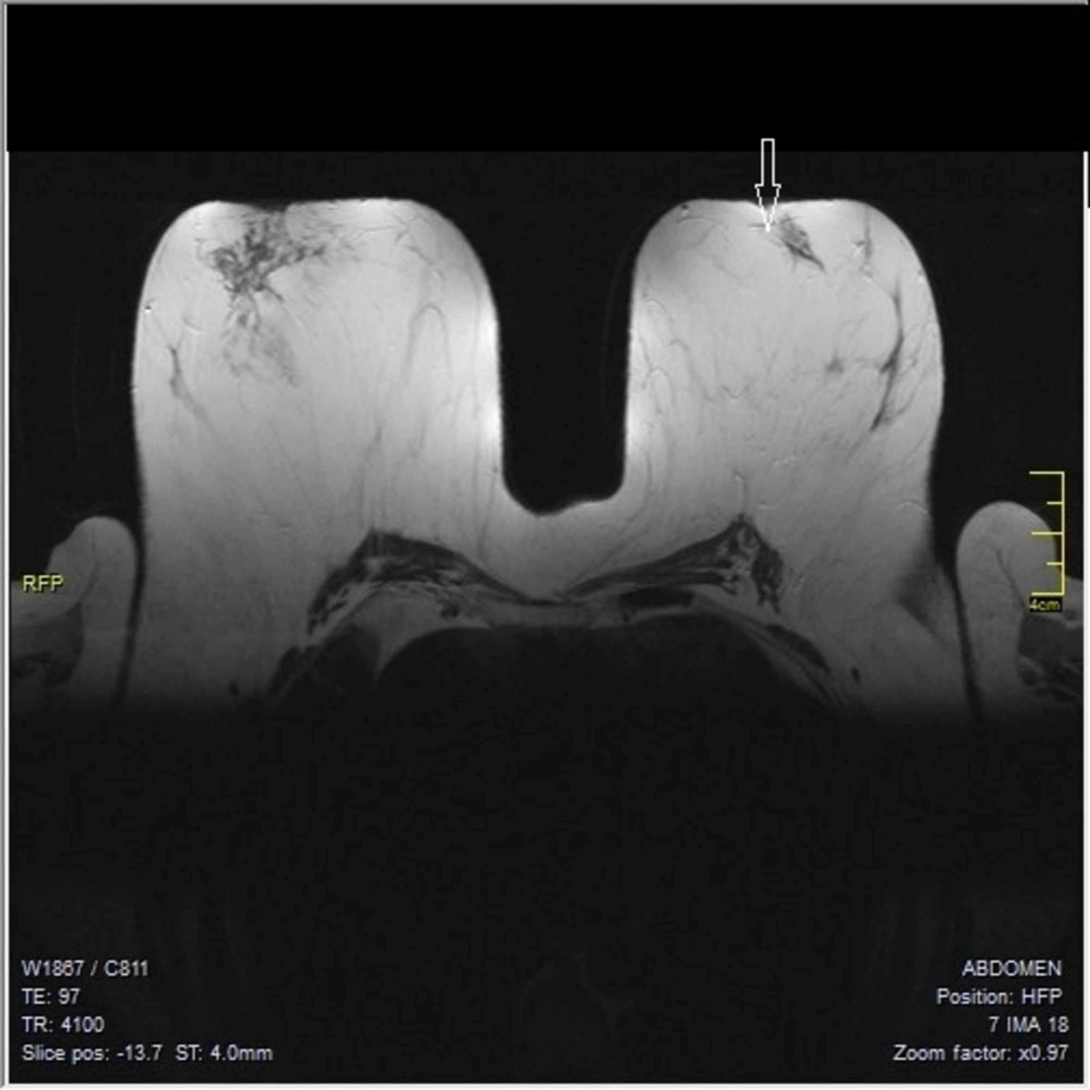
Magnetic resonance mammography (MRM) demonstrating a linear lesion, mammography and ultrasound (US) invisible

Subsequently, full staging with computed tomography with contrast medium of the thorax and the upper abdomen, transvaginal ultrasonography, and full body Technetium 99m-methyl diphosphonate (99mTc MDP) bone scintigraphy was performed, with all results negative for metastases. Since breast ultrasound, digital mammography or CESM had not revealed any lesions, a breast magnetic resonance imaging (MRI) was ordered for further investigation. On the left breast, at the outer and upper quadrant, at the 2nd surgical hour behind the nipple along a milk duct, an irregular not circumscribed linear lesion was detected measuring 23 mm, with increased contrast enhancement as can been seen in Figure 5 and Figure 6. There were also reported numerous 1st and 2nd level lymph nodes in the left axilla with maximal diameter of 12 mm. No abnormal findings were noticed on the right breast, the right axilla and the supraclavicular regions. Breast MRI was categorized as category B according to American College Of Radiology Classification (ACR B) and as Magnetic Resonance Mammography Breast Imaging Reporting and Data System category 2 (MRM-BIRADS 2) for the right breast and MRM-BIRADS category 4c for the left breast.

After a multidisciplinary consultation, the patient was advised for MRI-guided wire localization lumpectomy and axillary dissection. However, the patient decided on undergoing mastectomy instead of lumpectomy, insisting for being treated as presenting with occult carcinoma of the breast. After informed and written consent was obtained, mastectomy and axillary dissection was performed. Two negative pressure drainages were placed, one in the axillary cavity and a second over the thoracic wall to avoid post-operative seroma or hematoma. Drainages were removed the second day post-operatively, there were no peri-operative complications and the wound healed well.

**Figure 7:**
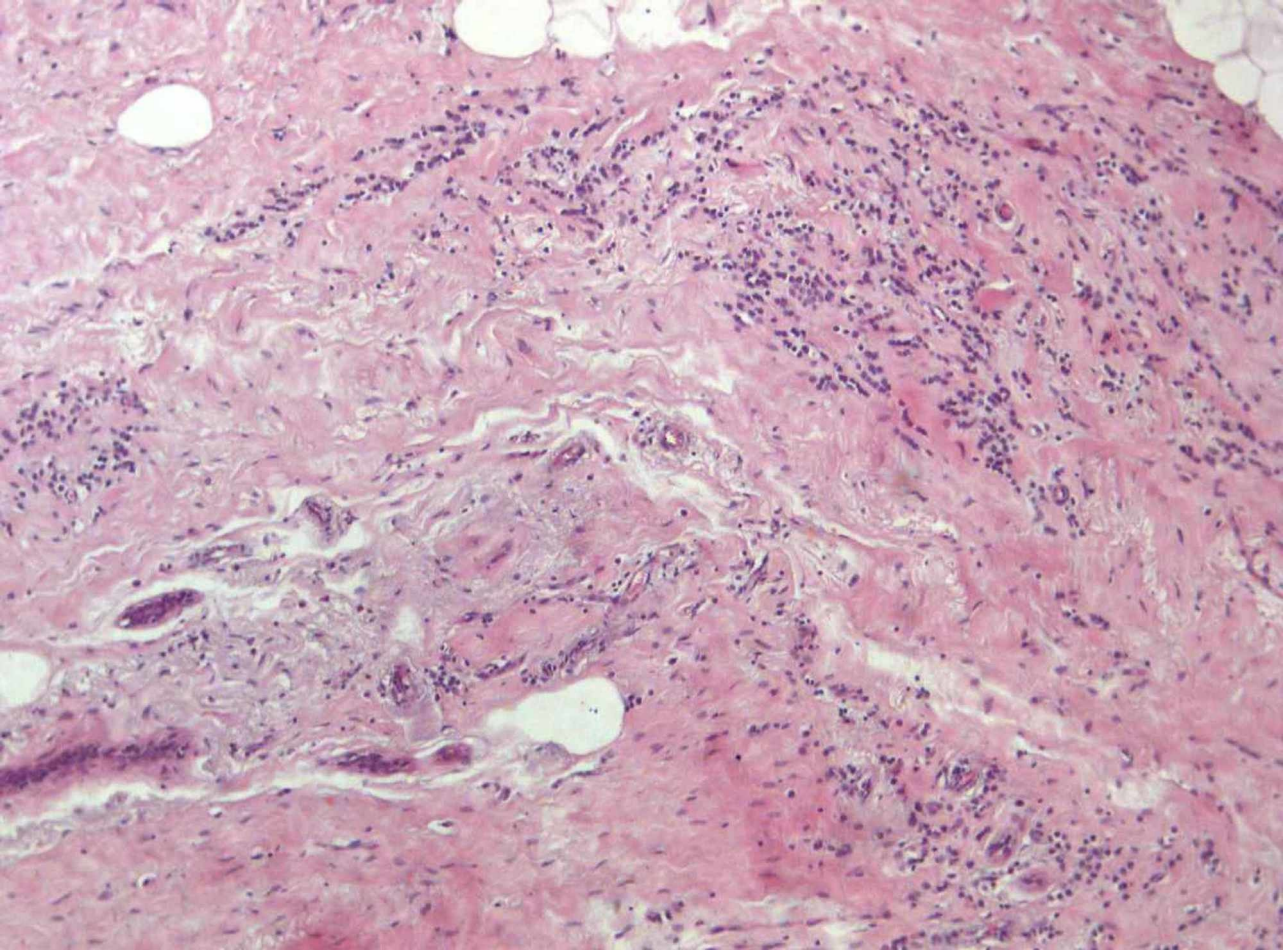
Breast invasive lobular carcinoma

Histopathology was suggestive of invasive lobular carcinoma throughout the whole extent of the breast parenchyma, as is demonstrated in Figure 7 with clear surgical margins . Pathologic staging was for the primary tumor stage 3 (T3) and for the regional lymph nodes stage 2a (N2a), since four out of the 17 excised lymph nodes were positive for metastatic infiltration.

## Discussion

Breast cancer (BC) is the most commonly diagnosed cancer and the leading cause of cancer related deaths among women in both developed and developing countries. Globally, the incidence rate of breast cancer has been on rapid increase over the past few decades [3]. Invasive breast cancer is a heterogeneous disease of two main histological types: invasive carcinoma of No Special Type or Not Otherwise Specified or Invasive Ductal Carcinoma (NST or NOS or IDC), and Invasive Lobular Carcinoma (ILC), with the first being by far the most common. During the last quarter of the past century, the incidence of ILC has increased especially among postmenopausal women, affecting about 20/100,000 women every year worldwide, whereas the incidence of IDC has remained the same [4]. This rise in incidence has been linked to the increasing use of menopausal hormone replacement therapy rather than to improved early detection. Advancing age and female sex are the most common risk factors for breast cancer. Several breast cancer risk factors preferentially promote ILC development: late age at first birth, early menarche, late age at menopause, BReast CAncer gene 2 (BRCA2) germline mutations and cadherin 1 (CDH1), the gene coding for the E-cadherin adhesion protein mutations [5].

ILCs are generally diagnosed at a more advanced stage, with an average age at diagnosis three years greater than IDC [6]. In clinical breast examination the findings are vague, such as thickening or induration found in ILC tumours, as opposed to a discrete nodule in IDC tumours. In patients with dense breast tissue or fibrocystic mastopathy, clinical examination and early diagnosis of ILC is particularly challenging. Therefore, it is reasoned that overall ILC is diagnosed in a more advanced stage. Often, patients present with larger tumor sizes and compared to grade-matched IDCs. In our case, the patient's first symptom was an enlarged axillary lymph node and this clinical presentation is related to the fact that ILCs are associated with a higher nodal stage, higher absolute number of positive nodes, and higher ratio of positive nodes [7]. They are also associated with a higher percentage of multifocal, multicentric, and bilateral cases, lower histological grade, higher rate of hormone receptor (ER/PR) positivity, lower rate of HER2 positivity, and low tumour cell proliferation index [8].

The reported sensitivity of mammography for the detection of ILC varies widely from 34% to 92% and is inversely related to breast density [9] and in the case reported above, mammography failed to reveal any findings related to malignancy. ILC is one of the most important reasons for false-negative mammograms. Normal or benign mammographic findings in ILCs are reported in 8 to 16% of cases and they may become clinically apparent within a period of 24 months after a negative mammography screening result. The imaging features of ILC are variable with a mass being the most common reported mammographic sign, followed by asymmetric opacities, architectural distortion, and ipsilateral decrease in breast size. No distinct mammographic sign exists, which allows prospectively to distinguish ILC from other types of breast cancer [10].

The sensitivity of ultrasound in the demonstration of ILC is reported between 68% to 98% and increases with the use of higher frequency probes [11]. The most common sonographic appearance of ILC is a hypoechoic mass with posterior acoustic shadowing, occurring in up to 60% of cases. In a further 21% there is an irregular mass without shadowing. Lobular tumours can also manifest merely as an area of posterior acoustic shadowing without an associated visibly distinct mass [12]. Finally, as in mammography, ILC can escape detection on sonographic investigation and remain invisible [13], as it is demonstrated in the case presentation as well.

The use of preoperative magnetic resonance imaging (MRI) has been controversial, due to concerns regarding false-positive results, leading to unnecessary surgical interventions. Its moderate specificity of 67.4% reduces its clinical usefulness. MRI has a sensitivity of 96% for the detection of ILC and even higher sensitivity was found by triple assessment (clinical evaluation, mammography with or without ultrasound and cytology or preferably histology), with false-negative rates reported low, but not zero [14]. The most common MRI presentation of ILC is that of a mass with irregular or spiculate margins, followed by a non-mass lesion in 20 to 40% of cases [15]. Current indications for MRI include work-up for occult breast cancer in the setting of clinically positive nodes [12].

Axillary lymph node status is an extremely important prognostic factor in the assessment of breast cancer patients. Although not always performed for axillary lymph node staging, core needle biopsy is a well-established procedure in case of suspicious axillary lymph nodes, to triage patients for sentinel node or axillary node dissection [16]. When negative surgical margins can be achieved, breast conservative surgery is preferred over mastectomy [17]. Although the role of axillary dissection seems to diminish as standard procedure of breast cancer treatment and staging, it is indicated in cases with node-positive disease at diagnosis and axillary lymphadenectomy is associated with improved survival in patients presenting with clinical N2-3 invasive breast cancer [18]. 

## Conclusions

In this case report we would like to clearly highlight that breast cancer should be definitely included in the differential diagnosis of enlarged axillary lymph nodes, even if there is no other clinical or radiographic presentation of breast disease. Furthermore, patients with ILC may present at an advanced stage of disease, even with minimal findings in clinical examination or imaging since ILC may not be detected in digital mammography or in breast ultrasound. Therefore, clinical examination and biopsy of any suspicious lesion must not be omitted.
